# The clinical journey of belantamab mafodotin in relapsed or refractory multiple myeloma: lessons in drug development

**DOI:** 10.1038/s41408-025-01212-0

**Published:** 2025-02-07

**Authors:** Pralay Mukhopadhyay, Hesham A. Abdullah, Joanna B. Opalinska, Prani Paka, Eric Richards, Katja Weisel, Suzanne Trudel, Maria-Victoria Mateos, Meletios Athanasios Dimopoulos, Sagar Lonial

**Affiliations:** 1 GSK, Upper Providence, PA USA; 2 University Medical Center of Hamburg-Eppendorf, Hamburg, Germany; 3 Princess Margaret Cancer Centre, Toronto, ON Canada; 4 University Hospital of Salamanca/IBSAL/CIC/CIBERONC, Salamanca, Spain; 5 Department of Clinical Therapeutics, School of Medicine, National and Kapodistrian University of Athens School of Medicine, Athens, Greece; 6 Winship Cancer Institute, Emory University Hospital, Atlanta, GA USA

**Keywords:** Drug development, Cancer

## Abstract

Patients with relapsed/refractory multiple myeloma (RRMM) have a poor prognosis and a need remains for novel effective therapies. Belantamab mafodotin, an anti–B-cell maturation antigen antibody-drug conjugate, was granted accelerated/conditional approval for patients with RRMM who have received at least 4 prior lines of therapy, based on response rates observed in DREAMM-1/DREAMM-2. Despite the 41% response rate and durable responses observed with belantamab mafodotin in the Phase III confirmatory DREAMM-3 trial, the marketing license for belantamab mafodotin was later withdrawn from US and European markets when the trial did not meet its primary endpoint of superiority for progression-free survival compared with pomalidomide and dexamethasone. This review reflects on key lessons arising from the clinical journey of belantamab mafodotin in RRMM. It considers how incorporating longer follow-up in DREAMM-3 may have better captured the clinical benefits of belantamab mafodotin, particularly given its multimodal, immune-related mechanism of action with responses deepening over time. A non-inferiority hypothesis may have been more appropriate rather than superiority in the context of a monotherapy versus an active doublet therapy. Further, anticipation of, and planning for, non-proportional hazards arising from response heterogeneity may have mitigated loss of statistical power. With the aim of improving the efficacy of belantamab mafodotin, other Phase III trials in the RRMM development program (DREAMM-7 and DREAMM-8) proceeded to evaluate the synergistic potential of combination regimens in earlier lines of treatment. The aim was to increase the proportion of patients responding to belantamab mafodotin (and thus the likelihood of seeing a clear separation of the progression-free survival curve versus comparator regimens). Protocol amendments reflecting DREAMM-3 learnings could also be implemented prospectively on the combinations trials to optimize the follow-up duration and mitigate risk. The wider implications of the lessons learned for clinical research in RRMM and in earlier treatment settings are discussed.

## Introduction

Treatment of multiple myeloma (MM) has evolved with increased understanding of its complex molecular pathophysiology [1, 2]. While effective options have emerged to improve survival outcomes for patients with MM [3], the disease remains incurable following progression on treatment with immunomodulatory drugs, proteosome inhibitors (PIs) and anti-CD38 monoclonal antibodies; triple-refractory patients have particularly poor prognoses [4, 5]. Urgent unmet need exists for therapies with novel mechanisms of action (MOAs) to overcome treatment resistance and improve outcomes in relapsed/ refractory MM (RRMM) [6, 7].

The antibody-drug conjugate (ADC) belantamab mafodotin comprises a humanized, afucosylated, anti–B-cell maturation antigen (BCMA) monoclonal antibody linked to the microtubule inhibitor monomethyl auristatin F (MMAF) to provide a unique MOA that combines an immunotherapy with a targeted therapy [8, 9]. Single-agent belantamab mafodotin was granted accelerated/ conditional approval by the United States Food and Drug Administration (FDA) and European Medicines Agency (EMA) in 2020 for patients with RRMM who have received at least 4 prior lines of therapy (LOTs; including an immunomodulatory agent, PI, and anti-CD38 monoclonal antibody). Approval was based on the overall response rates (ORRs) observed in the Phase I DREAMM-1 (NCT02064387) and Phase II DREAMM-2 (NCT03525678) clinical trials [10–13]. The subsequent Phase III, confirmatory DREAMM-3 (NCT04162210) study of belantamab mafodotin monotherapy versus pomalidomide with dexamethasone in patients with RRMM who had received two or more prior LOTs did not meet the primary endpoint of superiority for progression-free survival (PFS) [14]. Due to not fulfilling confirmatory requirements, belantamab mafodotin was withdrawn from the US and Switzerland in 2023 and from the European Union and United Kingdom markets in 2024 [15, 16]. Nevertheless, belantamab mafodotin continues to be investigated in other Phase III trials as a treatment for patients with RRMM, in combination with standard of care (SoC) therapies [17–19].

**Fig. 1:**
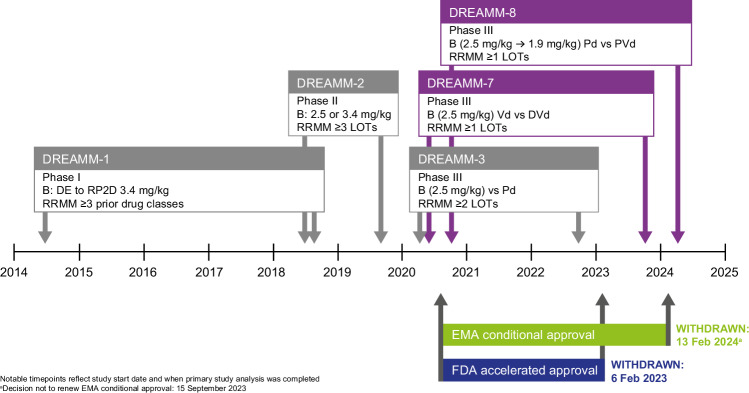
Overview of the belantamab mafodotin Phase II/III clinical development program timeline for key studies in RRMM. B belantamab mafodotin, DE dose-escalation, DVd daratumumab, bortezomib and dexamethasone, EMA European Medicines Agency, FDA Food and Drug Administration, LOT line of therapy, Pd pomalidomide and low-dose dexamethasone, PVd pomalidomide, bortezomib and dexamethasone, RP2D recommended Phase II dose, RRMM relapsed refractory multiple myeloma, Vd bortezomib and dexamethasone.

Here, we reflect on key lessons arising from the clinical development of belantamab mafodotin: 1) how to optimize study designs, duration of follow-up, and statistical approaches to better assess efficacy benefits resulting from its multimodal MOA (including consideration of combination therapy); 2) how to interpret data and apply learnings to the next steps of development; and 3) how a multi-pronged clinical development approach can facilitate continued drug discovery while bolstering the robustness of study designs, as illustrated by the Phase III DREAMM-7 and DREAMM-8 trials [17, 18]. We offer learnings for oncology drug development from a rare regulatory situation, where an approved drug was withdrawn from the market but continues to be investigated in the same disease in earlier treatment lines and with different or updated study designs (see Fig. 1 for an overview of relevant studies).

## Harnessing the MOA of belantamab mafodotin

### Multimodal MOA

BCMA, a member of the tumor necrosis factor receptor superfamily (TNFRSF17), is an attractive therapeutic target in MM, due to high surface expression on malignant plasma cells but not on normal tissues [8, 9]. Belantamab mafodotin binds to BCMA to enact a multimodal MOA: following cellular antibody internalization, the cytotoxic payload (MMAF) is released and disrupts the intracellular microtubule network, causing MM cell cycle arrest and apoptosis of MM cells [8]. Afucosylation improves antibody binding to FcγRIIIa to enhance antibody-dependent cellular cytotoxicity (ADCC) and phagocytosis (ADCP) [8, 9]. Additionally, immunogenic cell death potentially enhances antigen presentation, resulting in durable adaptive immune response and immunologic memory; the initial induction of immunogenic cell death requires BCMA binding and toxin release [9].

### Linking MOA with efficacy

The proportional contribution of each component of the MOA to the efficacy outcomes observed is not fully understood; however, preclinical studies indicate that belantamab mafodotin targets both dividing (via antibody-drug conjugate) and non-dividing (via ADCC/ADCP) tumor cells [20]. This stimulates tumor regression and enhances durability of response via its direct cytotoxic effects (while augmenting intratumor immune cell infiltration), and by engaging the adaptive immune response and immunologic memory [9]. In vitro and in vivo studies suggest that belantamab mafodotin efficacy is characterized by higher T-cell and natural killer lymphocyte numbers and increased markers of T-cell activation and immune-mediated tumor cell death [7, 9].

Although membrane-bound and soluble BCMA levels are consistently elevated in patients with newly diagnosed and RRMM, it is unclear how BCMA expression correlates with response to treatment [21–23], and no useful biomarker predictive of the clinical benefit of belantamab mafodotin has been identified. There is also considerable variability in BCMA expression between MM samples, likely owing to different study methodologies and the heterogeneity of disease [21].

Currently, resistance mechanisms to belantamab mafodotin remain unclear. One possibility is BCMA target loss, a rare event that may contribute to MM resistance to anti-BCMA chimeric antigen receptor T-cell or bispecific T-cell engager therapies [24, 25]. Additionally, a recent report demonstrated BCMA mutations following those treatments, but samples from patients targeted with belantamab mafodotin were not included in this analysis [25]. Further investigations of resistance mechanisms are actively being researched.

Patients who are sensitive to belantamab mafodotin generally respond quickly, likely due to rapid release of the cytotoxic payload (MMAF) [8, 26]. Often, responses show deepening over time, even during dose delays instituted to manage ocular events [14, 27]. This pattern of response suggests immune-related mechanism involvement, which may continue after the last administration of belantamab mafodotin prior to the dose delay, or after the end of treatment.

## Learnings from monotherapy treatment with belantamab mafodotin

Belantamab mafodotin represents a novel therapy for patients with RRMM [8, 28] who often have a dismal prognosis [4]. The poor outcomes of patients with RRMM were highlighted by the Monoclonal Antibodies in Multiple Myeloma: Outcomes after Therapy Failure (MAMMOTH) study, which retrospectively analyzed the natural history and outcomes of 275 patients across 14 US centers. Median OS was only 8.6 months, ranging from 11.2 months for patients who were not simultaneously refractory to an immunomodulatory drug and PI, to 5.6 months for patients refractory to anti-CD38 monoclonal antibody, two immunomodulatory drugs and two PIs (‘penta-refractory’) [4]. LocoMMotion – which evaluated the effectiveness of SoC in 248 triple-class exposed patients – reported similarly bleak findings (median OS: 12.4 months) [29]. These poor outcomes signpost the clinical importance of any agent demonstrating substantive clinical activity in this setting, particularly for patients who are triple-class exposed or refractory.

### Response rates and duration of response (DOR)

Belantamab mafodotin monotherapy is efficacious in terms of response to treatment in patients with RRMM, including heavily pretreated patients. In the Phase I DREAMM-1 trial (where 97% of patients were refractory to a PI and 94% to an immunomodulatory drug, and 37% had also received an anti-CD38 monoclonal antibody), the ORR with belantamab mafodotin was 60% and median DOR was 14.3 months [30]. Of note, the ORR for patients previously treated with daratumumab, an anti-CD38 antibody, was 43% [30].

In the Phase II DREAMM-2 trial, eligible patients had received ≥ 3 prior LOTs and all were triple-class refractory (i.e., to an immunomodulatory drug, PI, and anti-CD38 antibody) in the belantamab mafodotin 2.5 mg/kg and 3.4 mg/kg dose groups. The final analysis, with a median follow-up of 12.5 months, showed that patients treated at 2.5 mg/kg had an ORR of 32% and median PFS of 2.8 (95% CI, 1.6–3.6) months. Of the treatment responders, 58% had a very good partial response or better [≥VGPR]; median PFS was 14.0 months (95% CI, 9.7–not reached) and median DOR was 12.5 months [27]. The ORR was 31% at 6.3 months’ median follow-up [28], indicating a sustained response over time. Patients who required an extended dose delay for adverse event management ( > 63 days, equivalent to three cycles) still experienced long response durations [27], a possible consequence of the immune-related aspect of the multimodal MOA of belantamab mafodotin.

Patients were more heavily pretreated in DREAMM-2 than in DREAMM-1 ( > 4 LOTs: 84% versus 57%), which may help explain the lower ORR observed in this Phase II trial. All patients (100%) treated with 2.5 mg/kg belantamab mafodotin were refractory to an immunomodulatory drug or PI, and daratumumab (compared with 37% of patients in DREAMM-1 who had received daratumumab and were refractory to the other drug classes) [28, 31].

The ORRs and durability of responses observed in DREAMM-1 and DREAMM-2 in patients with RRMM [27, 28, 30, 31] supported Phase III investigation of belantamab mafodotin monotherapy in the DREAMM-3 trial, in which eligible patients had progressed on at least two prior LOTs (including the immunomodulatory drug lenalidomide, and a PI), and 42% of those treated with belantamab mafodotin had previously received anti-CD38 antibodies. Randomization was stratified by previous anti-CD38 therapy (associated with a poor prognosis [4]), International Staging System stage, and number of previous therapies, to ensure balance between study arms in disease severity and prior treatment exposure/refractoriness. Results from DREAMM-3 are summarized in Table 1. The ORR with belantamab mafodotin monotherapy was 41% (versus 36% for pomalidomide and dexamethasone); 62% of the responders to belantamab mafodotin achieved ≥VGPR and median DOR was not reached [14]. An updated analysis with approximately 18 months of additional follow-up from the primary analysis confirmed the depth and durability of response was maintained with belantamab mafodotin (the ORR remained the same as the primary analysis: 41% versus 36% for pomalidomide and dexamethasone, with a median DOR of 24.9 versus 10.4 months, respectively) (Table 1) [32].

**Table 1:** Patient populations, treatment arms and efficacy outcomes of the Phase III DREAMM-3, DREAMM-7, and DREAMM-8 trials.

Patient population	DREAMM-3				DREAMM-7		DREAMM-8	
	Primary Analysis [14]		Updated Analysis [32]		Interim Analysis [18]		2nd Interim Analysis [17]	
	RRMM with ≥2 prior LOTs including immunomodulatory drug and PI				RRMM with ≥1 prior LOT but not refractory to anti-CD38 therapy		RRMM with ≥1 prior LOT including lenalidomide	
Treatment arms	Belantamab mafodotin 2.5 mg/kg Q3W (*n* = 218)	Pd (*n* = 107)	Belantamab mafodotin 2.5 mg/kg Q3W (*n* = 218)	Pd (*n* = 107)	Belantamab mafodotin 2.5 mg/kg Q3W + Vd (*n* = 243)	DVd (*n* = 251)	Belantamab mafodotin 2.5 mg/kg Q4W for Cycle 1 then 1.9 mg/kg Q4W from Cycle 2 onwards + Pd (*n* = 155)	PVd (*n* = 147)
*Efficacy*								
Median (range) follow-up, mo	11.5 (5.5–17.6)	10.8 (5.6–17.1)	22.4 (0.6–43.0)	21.9 (0.0–44.2)	28.2 (0.1–40.0)		22.4 ( < 0.1–36.4)	20.5 (0.1–39.2)
PFS								
Median (95% CI), mo	11.2 (6.4–14.5)	7.0 (4.6–10.6)	–	–	36.6 (28.4–NR)	13.4 (11.1–17.5)	NR	12.7 (9.1–18.5)
PFS rate	–	–	–	–	69% at 18 mo	43% at 18 mo	71% at 12 mo	51% at 12 mo
HR (95% CI); *P*-value	1.03 (0.72–1.47); *P* = 0.56		0.86 (0.63–1.18)		0.41 (0.31–0.53); *P* < 0.001		0.52 (0.37–0.73); P < 0.001	
OS								
Median (95% CI), mo	21.2 (18.7–NR)^a^	21.1 (15.1–NR)^a^	–	–	33.9 (21.9–NR)^b^	15.2 (12.3–21.1)^b^	19.0 (12.2–23.3)^c^	12.7 (8.0–18.5)^c^
OS rate	–	–	–	–	84% at 18 mo	73% at 18 mo	83% at 12 mo	76% at 12 mo
HR (95% CI); *P*-value	1.14 (0.77–1.68); *P* = 0.75		0.93 (0.69–1.26)		0.57 (0.40–0.80)		0.77 (0.53–1.14)	
ORR, %	41	36	41	36	83	71	77	72
sCR/CR	10	3	–	–	35	17	40	16
≥ VGPR	25	8	–	–	66	46	64	38
PR	16	28	–	–	17	25	14	34
CBR ( ≥ MR)	47	47	–	–	86	76	80	78
Median (range) duration of response, mo	NR (17.9–NR)	8.5 (7.6–NR)	24.9 (20.7–32.5)	10.4 (7.6–20.0)	35.6 (30.5–NR)	17.8 (13.8–23.6)	–	–

^a^Data on OS were 38% immature at data cutoff (September 12, 2022).

^b^Data on OS were 29% mature at cutoff (October 2, 2023). 25^th^ percentile of the distribution of OS duration is shown; follow-up for OS is ongoing.

^c^Data on OS were 35% mature (as of January 29, 2024). 25^th^ percentile of the distribution of OS duration is shown; follow-up for OS is ongoing.

*CBR* clinical benefit rate, *CI* confidence interval, *CR* complete response, *DVd* daratumumab, bortezomib and dexamethasone, *HR* hazard ratio, *LOT* line of therapy, *mo* months, *MR* minimal response, *NR* not reached, *ORR* overall response rate, *OS* overall survival, *Pd* pomalidomide and dexamethasone, *PFS* progression-free survival, *PI* proteosome inhibitor, *PR* partial response, *PVd* pomalidomide, bortezomib and dexamethasone, *Q3W* every 3 weeks, *Q4W* every 4 weeks, *RRMM* relapsed/refractory multiple myeloma, *sCR* stringent complete response; *VGPR* very good partial response.

While cross-trial comparisons are difficult, particularly given the evolution of SoC therapies for RRMM in recent years, the response data for belantamab mafodotin monotherapy are positive in the context of published data for combination regimens in RRMM. The belantamab mafodotin monotherapy trials included a relatively high proportion of patients who were heavily pretreated and triple-refractory versus trials of triple versus doublet combinations in RRMM (Table 2) [33–54]. However, ORRs and VGPRs observed with belantamab mafodotin monotherapy in DREAMM-2 and DREAMM-3 (Table 1) [14, 27, 28, 32] are comparable to those for doublet regimens in the control arms of the triplet trials [33–54], with superior durability of response (Table 2), particularly in the third- and later-line settings.

**Table 2:** Pivotal Phase II/III trials of triplet versus doublet combination regimens in RRMM: patient populations, prior therapies and summary of efficacy.

Trial/regimen	Prior LOTs, median (range)	Prior PI /refractory to PI, %	Prior IM drug / refractory to IM drug, %	Prior anti-CD38 therapy, %	Median follow-up, mo	ORR, %	≥VGPR, %	Median DOR, months	Median PFS, months	Median OS, months
***RRMM 2*** ***L or 3*** ***L***										
**CANDOR**^**a**^ **[**36, 52]										
KDd (*n* = 312)	2(1,2)	93 / 28 (bor)	66 / 32 (to len)	< 1	17.2	84	69	NE	NR	NR
Kd (*n* = 154)	2(1,2)	90 / 31 (bor)	71 / 36 (to len)	0	17.1	75	49	16.6	15.8	NR
**OPTIMISSMM** [49]										
PVd (*n* = 281)	2(1,2)	75 / 13	100 / 72	—	15.9	82	53	13.7	11.2	-
Vd (*n* = 278)	2(1,2)	77 / 13	100 / 69	—		50	18	10.9	7.1	-
**BOSTON** [42]										
SVd (*n* = 195)	2(1–3)	69 (bor) / —	39 (len) / —	6	13.2	76	45	20.3	13.9	NR
Vd (*n* = 207)	2(1–3)	70 (bor) / —	37 (len) / —	3	16.5	62	32	12.9	9.5	25
***RRMM 2*** ***L/3*** ***L*** +										
**TOURMALINE-MM** [46, 48]										
IRd (*n* = 360)	1(1–3)	69 / 1	54 / 21	—	14.7 (85 for OS)	78	48	20.5	20.6	53.6
Rd (*n* = 362)	1(1–3)	70 / 2	56 / 25	—		72	39	15	14.7	51.6
**POLLUX** [34, 35]										
DRd (*n* = 286)	1(1–11)	86 / 20	55 / 3	—	13.5 (79.7 for OS)	93	76	NR	NR	67.6
Rd (*n* = 283)	1(1–8)	86 / 16	55 / 4	—		76	44	17.4	18.4	51.8
**ASPIRE** [54]										
KRd (*n* = 396)	2(1–3)	66 (bor) / —	20 (len) / —	—	32.3	87	70	28.6	26.3	NR
Rd (*n* = 396)	2(1–3)	66 (bor) / —	20 (len) / —	—	31.5	67	40	21.2	17.6	NR
**ELOQUENT-2**^**b**^ [39, 44]										
ERd (*n* = 321)	2(1–4)	68 (bor)	53 / —	—	24.5 ( ≥ 70.6 for OS)	79	33	21	19.4	48.3
Rd (*n* = 325)	2(1–4)	71 (bor)	54 / —	—		66	28	17	14.9	39.6
**IKEMA** [45, 53]										
IKd (*n* = 179)	2(1–4)	93 / 31	76 / 44	1	20.7 (56.6 for OS)	87	73	—	NR	NR
Kd (*n* = 123)	2(1–4)	85 / 36	81/ 47	0		83	56	—	19.2	50.6
**APOLLO** [40, 41]										
DPd (*n* = 151)	2(1–5)	100 / ^c^	100 / ^c^	—	16.9 (39.6 for OS)	69	51	NR	12.4	34.4
Pd (*n* = 153)	2(1–5)	100 / ^c^	100 / ^c^	—		46	20	15.9	6.9	23.7
**CASTOR** [47, 51]										
DVd (*n* = 251)	2(1–9)	67 / —	71 / —	—	7.4 (72.6 for OS)	83	59	NR	NR	49.6
Vd (*n* = 247)	2(1–10)	70 / —	80 / —	—		63	29	7.9	7.2	38.5
***RRMM 3*** ***L*** +										
**ICARIA-MM** [33, 50]										
IPd (*n* = 154)	3(2–4)	100 / 77	100 (len) / 96	—	11.6 (35.3 for OS)	60	32	13.3	11.5	24.6
Pd (*n* = 153)	3(2–4)	100 / 75	100 (len) / 94	—		35	9	11.1	6.5	17.7
**ELOQUENT-3** [37, 38]										
EPd (*n* = 60)	3(2–8)	100 (bor) / 78	98 / 90 (len)	2	≥ 9.1 ( ≥ 45 for OS)	53	20	NR	10.3	29.8
Pd (*n* = 57)	3(2–8)	100 (bor) / 82	100 / 84 (len)	4		26	9	8.3	4.7	17.4

^a^Updated analysis with median follow-up of 27.8 months (KDd) and 27.0 months (Kd): median PFS 28.6 months for KDd versus 15.2 months for Kd;

ORR 84% versus 73% and ≥VGPR 69% versus 47% [52].

^b^Median DOR for updated analysis: 21.9 months for ERd and 17.9 months for Rd [39].

^c^All patients received either a PI or an IM drug. Refractory to PI: 47% for DPd versus 49% for Pd. Refractory to IM drug: 79% for DPd versus 80% for Pd [40].

2 L, second line; 3 L, third line, *bor* bortezomib, *DOR* duration of response, *DPd* daratumumab, pomalidomide and dexamethasone, *DRd* daratumumab, lenalidomide and dexamethasone; *DVd* daratumumab, bortezomib and dexamethasone, *EPd* elotuzumab, pomalidomide and dexamethasone, *ERd* elotuzumab, lenalidomide and dexamethasone, *IKd* isatuximab, carfilzomib and dexamethasone, *IM* immunomodulatory, *IPd* isatuximab, pomalidomide and dexamethasone, *IRd* ixazomib, lenalidomide and dexamethasone, *Kd* carfilzomib and dexamethasone, *KDd* daratumumab, carfilzomib, and dexamethasone, *KRd* carfilzomib, lenalidomide and dexamethasone, *len* lenalidomide, *LOTs* lines of therapy, *NE* not estimable, *NR* not reached, *ORR* overall response rate, *OS* overall survival, *Pd* pomalidomide and dexamethasone, *PFS* progression-free survival, *PI* proteosome inhibitor, *PVd* pomalidomide, bortezomib and dexamethasone, *Rd* lenalidomide and dexamethasone, *RRMM* relapsed/refractory multiple myeloma, *SVd* selinexor, bortezomib and dexamethasone, *Vd* bortezomib and dexamethasone; *VGPR* very good partial response.

### PFS result in DREAMM-3

Median PFS in DREAMM-3 was numerically longer at 11.2 months for belantamab mafodotin compared to 7.0 months for pomalidomide and dexamethasone; however, the overall difference between arms in the risk of disease progression or death did not reach statistical significance at the primary analysis (hazard ratio, 1.03; 95% CI, 0.72–1.47; *P* = 0.56) [14]. This was likely driven by the numerically higher number of progression events at earlier timepoints in the belantamab mafodotin arm, following which the risk of disease progression or death was higher in the pomalidomide and dexamethasone arm. In hindsight, there are several associated learnings, as described below.

#### Impact of heterogeneity of response and duration of follow-up

The Kaplan–Meier analysis of PFS in DREAMM-3 suggests that the ORR of 41% in the belantamab mafodotin arm was not sufficient to demonstrate statistical superiority for PFS over pomalidomide and dexamethasone at an aggregate population level [14]. Overall, the data indicate heterogeneity in the treatment effect of belantamab mafodotin. The 41% of patients who responded often achieved VGPR or better (25%), resulting in long DOR and PFS [14]. The depth of response was further supported by the achievement of minimal residual disease (MRD) negativity in 8% of patients receiving belantamab mafodotin, but none in the pomalidomide and dexamethasone arm at ~22 months of follow-up [32]. Unfortunately, the remaining 59% of patients who had no response to belantamab mafodotin at the time of the primary analysis progressed quickly [14]. In the pomalidomide and dexamethasone arm of DREAMM-3, the response pattern was slightly different: although a smaller proportion (8%) of patients had VGPR or better within the 36% of patients who responded, the remaining 28% of responders achieved partial response (and an additional 11% had minimal response) [14]. Dexamethasone is also known to inhibit monoclonal protein secretion and bolsters the efficacy of some drug combinations [55], which may have prevented progression in some patients who had stabilization of their disease. While it is difficult to specifically pinpoint the precise cause of the slightly higher risk of disease progression in the first 3 months in the belantamab mafodotin arm of DREAMM-3 (as shown by the Kaplan–Meier curve for PFS), the above factors, along with statistical probability considerations during treatment arm allocation and chance factors affecting progression, may have played a role.

The observed response heterogeneity in DREAMM-3 resulted in violation of the proportional hazards assumption, with early crossing of the Kaplan–Meier curves for PFS at around 3 months (due to early treatment discontinuations arising from progression in a subset of patients), before the separation in favor of belantamab mafodotin could be observed. No specific clinical characteristics at baseline were identified that would explain the larger proportion of patients who progressed early in the belantamab mafodotin arm. However, of the patients who progressed within the first 3 months, those in the belantamab mafodotin arm had a higher number of prior LOTs and a shorter time from diagnosis compared with the pomalidomide and dexamethasone arm, indicating a more aggressive disease trajectory for some patients [14].

The final PFS analysis in DREAMM-3 was planned to be conducted after observing approximately 151 events and follow-up of ≥4 months in all enrolled patients, but this timepoint was selected without a clear understanding of the response mechanisms that would expect to lead to PFS outcomes improving in favor of belantamab mafodotin over time. The hazard ratio for PFS lowered to 0.86 (95% CI, 0.63–1.18) with the additional follow-up. The same trend was also observed for OS, where the hazard ratio reduced to 0.93 (95% CI, 0.69–1.26) (Table 1) [14, 32]. This numerical improvement in the PFS and OS treatment effect can be attributed to the long durability of response in the belantamab mafodotin-treated patients [32].

#### Choice of comparator and statistical approach

It is possible that the choice of comparator may have contributed to the failure of DREAMM-3 to reach its primary endpoint of PFS. The combination of pomalidomide with dexamethasone was deemed an appropriate choice as a guideline-recommended and globally adopted SoC treatment regimen for patients with RRMM at the time of study design [14, 33, 37, 56]. However, comparing belantamab mafodotin monotherapy with doublet SoC therapy for RRMM was ambitious because the trial was designed to demonstrate superiority of a single agent over a doublet regimen, rather than non-inferiority. The Phase III FOCUS study of carfilzomib versus low-dose corticosteroids with cyclophosphamide is another study of patients with advanced RRMM that failed to demonstrate superiority for an investigational monotherapy versus an active doublet control regimen [57]. The absence of biomarkers helping to identify patients who are likely to respond to novel therapies [58] means that add-on designs or head-to-head non-inferiority comparisons tend to be pursued [59]. Other pivotal trials in RRMM have mainly compared outcomes for triplet (add-on) versus doublet regimens [5], rather than monotherapy versus doublet therapy. It is not known at this point if the addition of dexamethasone to the belantamab mafodotin arm may have led to better disease control in rapidly progressing patients and created a fairer comparison between doublet regimens. In addition, designing DREAMM-3 to test for a non-inferiority hypothesis before superiority could have led to a different outcome, as a loss of statistical power can occur from the failure to take into consideration the possibility of observing a non-proportional hazards treatment effect during study planning [60, 61].

#### Clinical interpretation in the context of MOA and safety considerations

Statistical power in DREAMM-3 could also have been improved through longer follow-up, considering what is now known about the multimodal MOA of belantamab mafodotin. It is well recognized that, compared with traditional cytotoxic and small-molecule drugs, targeted monoclonal antibody therapies with immune-related MOAs, belantamab mafodotin, and immuno-oncology (IO) agents such as immune checkpoint inhibitors (ICIs) have distinct pharmacokinetic and pharmacodynamic profiles [62], and unique efficacy kinetics that differ from traditional treatments [63]. Belantamab mafodotin shares some similarities with ICI efficacy kinetics given the immune-related efficacy demonstrated, and this should be taken into account for clinical trial design and the appropriate selection of endpoint assessments [64]. Experience from clinical trials of IO agents suggests that positive effects of treatment are appreciated through durability of response and the tail end of the PFS and OS curve [64–66]. Survival curves obtained with ICIs, such as anti-programmed cell death protein-1 therapies, sometimes show a detrimental early effect when compared to chemotherapy or other targeted agents before showing a treatment benefit; longer-term, the tail flattens, identifying a survival plateau of patients who achieve deep and durable responses [64]. Longer and durable responses have been evident from the tail of the curves plateauing in belantamab mafodotin-treated patients, which could be indicative of the immune-related aspect of the MOA. This was seen in the Kaplan-Meier PFS curve for belantamab mafodotin in DREAMM-3, which had a steeper gradient over the first 3 months than that for pomalidomide plus dexamethasone, while at later timepoints (19–23 months), PFS with belantamab mafodotin flattened to approximately 40% [14]. If a chemotherapy or cytotoxic payload is going to be efficacious, this is likely to happen soon after administration, but the MOA of IO agents leads to sustained anti-tumor effects over time, and improvement of relative efficacy with longer follow-up. Supporting this, DREAMM 3 continued to show improvement in treatment effect with additional follow-up from the primary readout.

It is also important to recognize that LOT can also impact the efficacy of cancer immunotherapies because of their MOA (i.e., to a greater extent than seen with purely cytotoxic agents such as chemotherapy), as a result of increasing T-cell exhaustion and tumor-driven immune escape, which may lead to therapeutic resistance and reduce response duration in later lines [67, 68]. IO agents including ICIs and ADCs are increasingly being investigated in earlier LOTs because of their potential to provide durable clinical benefit in this setting [68, 69].

Ocular adverse events (AE) such as dry eye and blurred vision are a recognized class effect of ADCs with an MMAF component [14, 28, 30, 70]. Although these events were reported with belantamab mafodotin monotherapy in the DREAMM-1, DREAMM-2 and DREAMM-3 trials [14, 28, 30], resulting discontinuation rates were very low (e.g., 2% in DREAMM-3) [14]. Dose modifications or extended dosing intervals provided effective management of ocular AEs and resolution during follow-up [14, 28, 30], without compromising efficacy (as shown by maintenance or deepening of responses) [14, 30]. A similar approach to management of ocular AEs was therefore taken in the belantamab mafodotin combination trials, as described later in this review.

#### Implications for clinical use and development of belantamab mafodotin

As discussed, for various reasons, the confirmatory Phase III study DREAMM-3 failed to show statistical superiority for the primary endpoint of PFS. This led to the withdrawal of belantamab mafodotin from US and European markets for patients with RRMM who received at least four prior LOTs including an anti-CD38 monoclonal antibody, immunomodulatory drug, and a PI [15, 16], thereby eliminating another potential option for patients at this late, and increasingly difficult, stage of their treatment journey. Conducting a non-inferiority trial (or requiring a longer follow-up to fully incorporate the benefit of the longer durability of response) may have asked a more appropriate clinical question given that the comparator of pomalidomide and dexamethasone was an active control and current SoC [71].

Despite the negative results of DREAMM-3, the development program for belantamab mafodotin continues to move forward and evolve in RRMM, taking on board some of the lessons learned from experience. The combination of belantamab mafodotin with other SoC therapies is currently being investigated to examine the extent to which synergistic effects are achieved by utilizing complimentary MOAs.

### Belantamab mafodotin combination trials in RRMM

#### Rationale: exploiting synergistic MOAs

Preclinical studies show that the MOA of belantamab mafodotin is enhanced by lenalidomide, pomalidomide, and bortezomib [8, 72], and the observed synergistic/additive effects support the rationale for combination therapy. Lenalidomide and pomalidomide are immunomodulatory drugs that increase interleukin-2 production in T-lymphocytes and decrease proinflammatory cytokine production; they also bind to an E3 ubiquitin ligase complex that leads to proteasomal degradation of two B-cell transcription factors, IKZF1 and IKZF3, killing MM cells [73]. In vitro, the ADCC activity of MMAF in belantamab mafodotin was enhanced in mononuclear immune cells pre-treated with lenalidomide [8], and in vivo, combination of the two agents synergistically inhibited tumor growth and provided a survival advantage in MM xenograft models compared with belantamab mafodotin monotherapy [72]. Synergistic anti-tumor activity in MM cells was also seen in combination with bortezomib [72], which inhibits the ubiquitin proteasome pathway (leading to degradation of intracellular proteins and apoptosis), induces immunogenic cell death through activation of the calreticulin pathways, and stimulates MM immunogenicity via activation of the cGAS/STING pathway and production of type I interferons [74, 75].

Combining belantamab mafodotin with other agents that have alternative targets and MOAs may also help circumvent the potential loss of efficacy caused by drug resistance [19], or the potential for antigen escape arising from acquired *TNFRSF17* biallelic deletions representing a shared, albeit rare, resistance mechanism to BCMA-directed therapies [25, 76].

#### Key combination trials

Belantamab mafodotin has been evaluated in combination with immunomodulatory drug and PI doublets in RRMM trials. In the Phase I/II Algonquin study of patients with RRMM (median 3 prior therapies [range 1–6] with 100% previously exposed to lenalidomide and a PI and 63% triple refractory), treatment with belantamab mafodotin at the recommended Phase II dose of 2.5 mg/kg in combination with pomalidomide and dexamethasone led to an ORR of 85.3%. The ≥VGPR rate was 75.7%, with an estimated 2-year PFS of 52.8% at a median follow-up of 13.9 months [77].

The Phase III DREAMM-7 and DREAMM-8 studies evaluated triplet regimens head-to-head in patients with RRMM, but in an earlier LOT than DREAMM-3 (as eligible patients had received ≥1 prior regimen). DREAMM-7 compared the combination of belantamab mafodotin, bortezomib and dexamethasone with daratumumab, bortezomib, and dexamethasone, whereas DREAMM-8 compared belantamab mafodotin, pomalidomide and dexamethasone with pomalidomide, bortezomib and dexamethasone [17, 18]. Although the timing of these trials overlapped with DREAMM-3, it was still possible to learn from experience and implement revisions to their study designs through protocol amendments, to avoid DREAMM-7 and DREAMM-8 undergoing a similar fate to DREAMM-3. The main change in both trials was to extend the follow-up for PFS (i.e., delaying the primary analysis to account for a greater number of events, and for the possibility of later separation of the PFS curves between arms [as seen in DREAMM-3]). Longer follow-up can also minimize loss in statistical power due to non-proportional hazards [60, 61]. In addition, to mitigate delay in development timelines, interim PFS analyses were introduced, to allow examination of the data at earlier timepoints (at a similar timepoint as the final analysis in the original protocol) and to assess if the treatment effect was positive. Interim analyses of DREAMM-7 and DREAMM-8 demonstrated robust efficacy with belantamab mafodotin in combination in patients with RRMM following ≥1 prior regimen, with greater depth of response, more durable responses, and significantly improved PFS versus comparator triplet regimens (summarized in Table 1). A recent update from DREAMM-7 demonstrated a statistically significant and clinically meaningful improvement in OS for belantamab mafodotin in combination with bortezomib and dexamethasone versus daratumumab in combination with bortezomib and dexamethasone [78]. At the time of the interim PFS analysis for DREAMM-8, the belantamab mafodotin regimen demonstrated a positive trend for improved OS, with follow-up ongoing for the next preplanned OS analysis. The results to date demonstrate that combining belantamab mafodotin with other agents and in earlier lines of treatment (compared with DREAMM-3) can improve efficacy outcomes in RRMM. As anticipated from the monotherapy trials, the belantamab mafodotin combination regimens utilized in DREAMM-7 and DREAMM-8 were associated with ocular AEs, but discontinuation due to these events remained low in the interim analyses (9%), and they were generally manageable with protocol-recommended dose and schedule modifications. Patients continue to derive benefit from treatment without negative impact on quality of life [17, 18, 79, 80].

## Conclusions

The primary endpoint of PFS superiority of belantamab mafodotin versus pomalidomide and dexamethasone was not met in DREAMM-3. This had dramatic clinical consequences for a patient population who have limited remaining therapeutic options, as it led to the withdrawal in the US and Europe of belantamab mafodotin as a treatment for patients with heavily-pretreated RRMM following at least 4 prior LOTs [15, 16]. However, the response data from DREAMM-3 showed that single-agent belantamab mafodotin had considerable clinical activity in this setting, with robust and durable responses consistent with the findings for triple-class refractory MM patients in DREAMM-1 and DREAMM-2 [14, 27, 30, 31, 81].

The failure to reach the primary endpoint of PFS is thought to be influenced by the observed heterogeneity in the treatment response (and subsequent durability of response) that affected both the outcome and interpretation of PFS in DREAMM-3. Heterogeneity is a major feature of MM and its disease course, influenced by the presence of high-risk features, renal impairment, extent of extramedullary disease, variance in the clonal evolution of MM cells/genetic aberrations, and the impact of exposure/acquired resistance to prior treatment, which may affect the timing of relapse [6, 82, 83]. Response to anti-BCMA therapy in RRMM may also correlate with baseline ’immune fitness’ (with improved outcomes reported for patients with lower levels of T-cell exhaustion or immunosuppressive regulatory T-cells) [23].

Suboptimal facets of the trial design and statistical approach also likely impacted DREAMM-3 outcomes. This is common to many prior confirmatory trials in oncology, where statistical or other study design challenges (including choice of regimen, comparator, or population) have led to underestimation of a drug’s true benefit [84]. A non-inferiority study hypothesis would have been more appropriate to evaluate belantamab mafodotin monotherapy with an SoC doublet in DREAMM-3. In addition, a longer observation time would have been preferable for comparing the two arms, to fully assess the long-term clinical benefit of belantamab mafodotin as a drug with a multimodal MOA that incorporates a cytotoxic payload and other antibody-mediated and immune-related processes. Further, anticipation of the non-proportional hazards effect and planning for this during trial design could have helped avoid a negative statistical outcome.

The results from DREAMM-3 also highlight the need to investigate multi-agent combination regimens in MM to address tumor heterogeneity and evaluate the potential of synergistic MOAs to improve efficacy (ideally, while making suitable adjustments in the SoC backbone dose and/or duration, to enable an optimal treatment schedule for patients). If a significant proportion of patients fail to experience a good response to treatment, this impacts the likelihood of seeing a clear separation of the PFS survival curves, supporting the rationale for strengthening the treatment regimen in earlier LOTs, where possible. DREAMM-7 and DREAMM-8 investigated the combination of belantamab mafodotin with other agents within alternative triplet regimens for RRMM following at least one prior LOT, and learnings from DREAMM-3 informed updates to the study designs, to help ensure better capture of efficacy. Similar learnings from other ‘failed’ trials in oncology have positively impacted the future design of clinical studies for agents that were subsequently approved, including IO/targeted treatments [85].

Trials of doublet and triplet regimens with a similar backbone in RRMM have typically been designed as add-on trials, to enable direct assessment of improved outcomes over SoC for agents that have an MOA different from that of established, effective treatments, and where synergies and additive effects might be demonstrated [6]. However, head-to-head, active control trials of drug efficacy and safety are believed, by many clinicians, to have the most informative design for their practice as it provides clarity on whether the experimental drug is better than SoC [58]. The head-to-head trial designs of DREAMM-7 and DREAMM-8 enabled direct comparison of triplet combination regimens with different MOAs, with positive results suggesting that belantamab mafodotin combinations have the potential to become a new SoC in patients at, or after, first relapse in RRMM. A statistically significant OS benefit was recently reported with belantamab mafodotin in combination with bortezomib and dexamethasone from DREAMM-7 [78], and while mature OS data are still awaited from DREAMM-8, a positive trend in OS with the belantamab mafodotin combination has already been observed [17]. Other large Phase III trials in RRMM (e.g., POLLUX, CASTOR, ELOQUENT-3) have shown that significant OS benefits can be demonstrated in patients receiving triplet regimens for a long period of time (i.e., with median follow-up of several years) [35, 38, 51]. However, the key difference to note is that these studies showed improvement over doublet therapy, while DREAMM-7 and DREAMM-8 are comparing two different triplet combinations.

Building on the success of DREAMM-7 and DREAMM-8, belantamab mafodotin continues to be investigated in newly diagnosed MM. For example, the ongoing Phase I DREAMM-9 (NCT04091126) study aims to evaluate belantamab mafodotin plus bortezomib, lenalidomide, and dexamethasone in patients with transplant-ineligible, newly diagnosed MM, and establish the recommended dose for future development of combinations in the first-line setting [86]. Results to date have shown high response rates ( ≥ 90%) and a manageable safety profile (including mitigation of ocular AEs with lower doses or extensions of the dosing interval) [86, 87]. Including additional surrogate endpoints, such as MRD, may be informative when established treatments are being assessed in an earlier LOT for MM, to help identify an initial clinical benefit that is correlated with long-term outcomes and traditional endpoints such as PFS and OS [88]. Higher rates of MRD negativity were seen in patients treated with belantamab mafodotin compared to the control in DREAMM-3 (8% versus 0% with pomalidomide and dexamethasone), DREAMM-7 (25% versus 10% with daratumumab, bortezomib and dexamethasone in patients achieving complete response or better) and DREAMM-8 (24% versus 5% with pomalidomide, bortezomib and dexamethasone for patients having complete response or better) [14, 17, 18]. These results demonstrate a considerable depth of response that could also provide benefit to patients with earlier-stage MM, including those receiving first-line treatment. On April 12, 2024, the FDA Oncologic Drugs Advisory Committee voted unanimously to allow MRD (at proposed timepoints of 9 and 12 months) as an accepted intermediate endpoint for accelerated approval in MM clinical trials, in both the newly diagnosed and RRMM settings [88].

Looking to the future, additional research is also warranted to identify when it might be best to use BCMA-targeted agents (in terms of LOT), and how to potentially sequence belantamab mafodotin with other anti-BCMA therapies approved for patients with RRMM (such as idecabtagene vicleucel, cilta-cel or teclistamab) [89]. When designing and conducting new trials, it will be important to bear in mind the lessons so far learned from the clinical journey of belantamab mafodotin in RRMM.

## Data Availability

Data used in the analysis were obtained from resources available in the public domain and previously published reports.
