# Correction: The clinical journey of belantamab mafodotin in relapsed or refractory multiple myeloma: lessons in drug development

**DOI:** 10.1038/s41408-025-01243-7

**Published:** 2025-03-26

**Authors:** Pralay Mukhopadhyay, Hesham A. Abdullah, Joanna B. Opalinska, Prani Paka, Eric Richards, Katja Weisel, Suzanne Trudel, Maria-Victoria Mateos, Meletios Athanasios Dimopoulos, Sagar Lonial

**Affiliations:** 1 GSK, Upper Providence, PA USA; 2 University Medical Center of Hamburg-Eppendorf, Hamburg, Germany; 3 Princess Margaret Cancer Centre, Toronto, ON Canada; 4 University Hospital of Salamanca/IBSAL/CIC/CIBERONC, Salamanca, Spain; 5 Department of Clinical Therapeutics, School of Medicine, National and Kapodistrian University of Athens School of Medicine, Athens, Greece; 6 Winship Cancer Institute, Emory University Hospital, Atlanta, GA USA

**Keywords:** Drug development, Cancer

Correction to: *Blood Cancer Journal* 10.1038/s41408-025-01212-0, published online 7 February 2025

In Figure 1, the trial periods and notable time points for the trials shown, have been amended to indicate the start dates and dates of data availability. The FDA and EMA withdrawal dates were also amended to indicate the dates of withdrawal rather than the dates of decision not to renew the accelerated/conditional approval. Additionally, for the DREAMM-8 trial, the BPd regimen was erroneously shown as BVd. This has been corrected and dosing of belantamab mafodotin has been updated. In Table 1 the DREAMM-3 primary analysis was clarified and some reference numbering was updated throughout the publication.
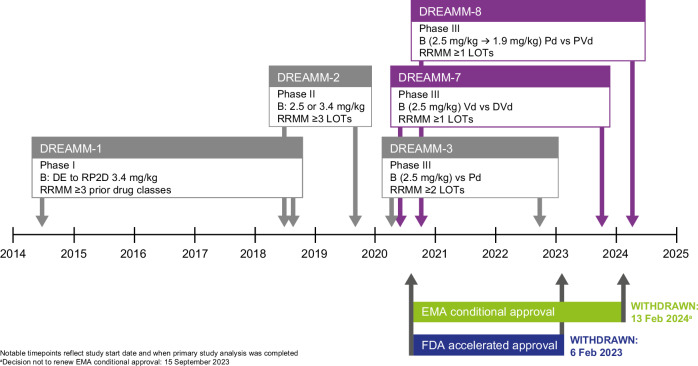


The original article has been corrected.

